# Persistent Papillary Membrane Associated with Atrophy of Choroid and Optic Nerve

**Published:** 1894-01

**Authors:** S. Latimer Phillips

**Affiliations:** Savannah, Ga.


### PERSISTENT PAPILLARY MEMBRANE ASSOCIATED WITH ATROPHY OF CHOROID AND OPTIC NERVE.

By S. LATIMER PHILLIPS, M. D.,
Savannah, Ga.

While to the anatomist and clinician the congenital defects of the human organism are extremely interesting, to the possessor they are not so agreeable, particularly as, in the majority of cases, they are incurable.

As all of the organs of the human body are exposed to these defects more or less, the eyes naturally come in for their share. These are made the more interesting from the latter-day anatomical and microscopical examinations.

In Germany such men as Manz. Deutschmann, C. Hess, Schweigger, Vossius, etc., have written most instructively upon this subject during recent years.

Of this class of congenital troubles none is more interesting than that peculiar condition known as persistent papillary membrane. It may take any form, from a thin cord across pupil, to complete occlusion of same. Recently a case of the former came under my observation, which I deem of sufficient interest to report. It is rather rare that we find these defects, then generally by accident. Most of the text-books pass this subject over without a word, but a few such as Noyes[1], Galezouski[2], Fuchs[3], Schmidt-Rimpler[4], give it a mere mention.

My case was that of a man thirty-three years of age who had consulted me for some throat trouble, and who incidentally mentioned that his vision in left eye was very poor. He was a splendidly developed man, in every way, save that of the eye.

His weight was one hundred and eighty pounds, with height of six feet. Irides gray. On superficial examination could detect nothing

[1] Diseases of the Eye.
[2] Traitê des Maladies des Yeux.
[3] Lehrbuch der Augenheilkunde.
[4] Augenheilkunde und Ophthalmoskopie.

wrong about eye. Since his earliest remembrance vision has been bad in left eye, and being aware of this never had eyes examined before, taking it as a matter of course. R. V.= $\frac{20}{xx}$ L.= $\frac{20}{c}$, with left eye can bear J. No. 12 with difficulty. On throwing light into pupil with ophthalmoscope, noticed thin dark line running diagonally across pupil which at first looked like a striation in lens. On more careful inspection this proved to be a thin thread inserted into the iris, just beyond the edge of the pupil, in the lesser iridic circle. The temporal attachment being just above the equatorial line, the nasal being about the middle and lower quadrant of iris. The anterior chamber was normal in depth ; the iris in its position. The pupil responded to light as any ordinary pupil would. The pupil readily dilated under the influence of a 4 per cent. sol. of cocaine, being perfectly round with no attachment to capsule of lens, or any irregularities whatever. The thread seemed to be elastic, and as pupil dilated seemed to stretch and become thinner. There was no other evidence of membrane in pupil. On looking at fundus found quite an extensive spot of central choroidal atrophy, with atrophy of upper third of papilla. Otherwise the fundus was normal. The atrophy of choroid and optic nerve might have been coincident with, or independent of the papillar membrane, but the fact that he had never had iritis or any inflammatory condition of the eye in his remembrance would lead us to believe that this was congenital also.

Some times the thread becomes attached to the posterior layer of the cornea, as in a case of much interest, reported by Prof. Vossius of Giesen[1]. A twelve year old boy suffering with blepharitis, peripheral, phlyctens and pannus of a scrofulous nature was brought to his clinic, where he remained under observation and treatment for many weeks.

He had intense photophobia, diffuse clouding of cornea with vascularization and roughening of epithelium, but no loss of substance. After clearing up of cornea, in its deeper layers, at center, was noticed a circumscribed, round, grayish white clouding size of a small pinhead, to which was attached the iris by a thin tense, long, gray brown thread. It had its origin in the tissue of the

Zur Kasuistik der Augeborenen Anomalies des Auges; *Beiträge Zur Augenheilkunde*, ix. Heft, S. 9.

iris near the papillary margin. The depth of outer chamber was normal and color of iris unchanged. The iris was in its natural position, and not drawn toward the cornea at all by the synechia. The pupil was round with good reaction and could be fully expanded by atropia, showing no adhesion between capsule and iris; in fact there was absence of all signs of synechia having been the sequel of an iritis or perforating ulcer of cornea. With the examination by the Zehender-Westien glass the gray-brown thread was found to consist of three small, short threads which sprung out of iris tissue in region of lesser circle; these little threads united themselves into a roundish gray tubercle, from which again sprung two very fine 2 to 3 m. long threads, which were inserted into the gray corneal cloud mentioned above. Without the glass these appear as one cord.

Samelson[1] reports a case somewhat similar. A young girl twenty years old, having some seventeen to eighteen of these threads joining iris to cornea.

In looking for the cause of these malformations, it is impossible to say whether they are due to some inflammatory condition during the uterine life analogous to those we find after birth, or to some unknown influence working during embryonic life, though often the latter would seem to be the more reasonable.

---

### TREATMENT OF DIPHTHERIA.

Drs. I. N. Love (*Medical Mirror*) and R. C. Atkinson have found that when the diphtheritic membrane is full-fledged or just developing, no better application for its dissolution can be made than the dry powder of papoid, applied by a camel-hair brush, moistened, thus taking up a liberal quantity, and applied directly or applied by insufflation. Repeated experiments with papoid applied to the membrane *in situ* resulted in its rapid softening, and a demonstration can be made of its value by taking a portion of the membrane, if separated from the mucous surface, and covering it with papoid. It will melt as does the snow under the morning sun.—*Medical Standard.*

[1] *Centralblat fur Augenheilk.* 1880. S. 215.

